# Nanofiltration of Mine Water: Impact of Feed pH and Membrane Charge on Resource Recovery and Water Discharge

**DOI:** 10.3390/membranes4020163

**Published:** 2014-03-27

**Authors:** Mark Mullett, Roberta Fornarelli, David Ralph

**Affiliations:** 1 Hatch Ltd., 144 Stirling St., 6000, Perth, Western Australia, Australia; E-Mail: mmullett@hatch.com.au; 2 School of Engineering and Information Technology, Murdoch University, South St., 6150, Murdoch, Western Australia, Australia; E-Mail: D.Ralph@murdoch.edu.au

**Keywords:** mine influenced water, nanofiltration, feed pH, iso-electric point, ion rejection, metal recovery, discharge criteria

## Abstract

Two nanofiltration membranes, a Dow NF 270 polyamide thin film and a TriSep TS 80 polyamide thin film, were investigated for their retention of ionic species when filtering mine influenced water streams at a range of acidic pH values. The functional iso-electric point of the membranes, characterized by changes in retention over a small pH range, were examined by filtering solutions of sodium sulphate. Both membranes showed changes in retention at pH 3, suggesting a zero net charge on the membranes at this pH. Copper mine drainage and synthetic solutions of mine influenced water were filtered using the same membranes. These solutions were characterized by pH values within 2 and 5, thus crossing the iso-electric point of both membranes. Retention of cations was maximized when the feed solution pH was less than the iso-electric point of the membrane. In these conditions, the membrane has a net positive charge, reducing the transmission rate of cations. From the recoveries of a range of cations, the suitability of nanofiltration was discussed relative to the compliance with mine water discharge criteria and the recovery of valuable commodity metals. The nanofiltration process was demonstrated to offer advantages in metal recovery from mine waste streams, concomitantly enabling discharge criteria for the filtrate disposal to be met.

## 1. Introduction

The management of water in mining operations is becoming increasingly scrutinized, with water reuse, water treatment and discharge being major issues faced by the industry [1]. Acid mine drainage (AMD) is a typical by-product of the mining industry and a specific type of mine influenced water (MIW). MIW is well known for its adverse impact on the environment and water security [2]. AMD occurs when rock containing reduced sulphur is exposed to air and water, resulting in metals and sulphate being released from a variety of rock types, and a broad range of metal concentrations and pH can result. Johnson and Hallberg [3] highlight two key points in the choice of suitable technologies to treat mine waters: (i) it is fundamental to consider mine water remediation as a resource, thus encouraging the recovery and recycling of the products of mine water treatment; and (ii) legislation defines discharge criteria that may determine the choice of a system to effectively remove sulphate, as well as metals and acidity from mine waters. 

Treatment of mine water is often seen as an end-of-pipe process aimed at producing a discharge stream that meets specified limits of acidity and concentrations of metals and sulphate. Extensive reviews have been published on treatment options for acid mine drainage and heavy metal containing wastewaters [3,4,5]. Lime neutralization and biological treatments are recognized as the traditional approaches [6]. Lime is added to precipitate some of the sulphate as gypsum and some metals as hydroxides. In biological treatments, anaerobic conditions are used to reduce the sulphate to sulphide, leading to the precipitation of metal sulphides that are incorporated in benthic organics and live biomass.

Ion exchange and membrane technologies are alternatives to treat mine waters [7]. Differing from lime neutralization and biological treatments, these technologies have the ability to not only remove potentially toxic metals and meet discharge criteria, but also to recover those metals and acid from the mine waters. Extraction of copper, nickel and cobalt from AMD by ion exchange has been already investigated and shown to produce positive net present values [8]. Membrane treatment by reverse osmosis (RO) and nanofiltration (NF) is also an established strategy for heavy metal removal, as it is capable of achieving strict discharge criteria, while providing high efficiency, easy operation and a low site “foot print” [4]. Recent studies successfully applied membrane separation to treat both synthetic and real mine water solutions [6,7,9,10,11]. The authors explored membrane performance under different experimental conditions with particular attention to the effects of solution temperature, operating pressure, feed flow and feed concentration on solute rejection and permeate flux. Relatively unexplored, however, is the effect of mine water pH on membrane performance, although its impact on solute rejections has been reported [6,9].

RO and NF are known to provide similar rejection performance for polluting metals [9,12]; however, NF has been suggested as the preferable treatment, because it has higher fluxes at lower pressure, leading to lower capital investment and lower cost of operation and maintenance [12]. NF also has the ability to selectively concentrate and recover commodity metals and sulphuric acid without concentrating the full total dissolved solids of the solution [11,13]. To demonstrate the economic advantage of NF over RO when treating MIW, an indicative cost analysis was recently performed by Fornarelli *et al.* [14]. It was shown that capital and operational costs for NF were about 10% and 30%, respectively, less than for RO. 

The NF separation mechanism can be identified as a sum of convection and diffusion transport mechanisms, *i.e.*, sieving effects, together with electromigration as a result of membrane charge [12,15]. In addition, the Donnan potential develops at the interfaces as a result of ion distribution [12]. Convective transport of ions with the water flux through the membrane is caused by the pressure difference between feed and permeate sides [15]. Similarly, diffusive transport is a consequence of the concentration gradient as achieved by the rejection of solutes [15]. Electromigration is caused by a “streaming potential” difference across the membrane. This streaming potential is caused by the electric current generated by the convective flow of a fluid that is necessarily charged through the pores of a charged membrane [15]. For uncharged molecules, sieving or size exclusion is primarily responsible for separation and is controlled by molecular size in solute form. For ionic species, both sieving and electromigration are responsible for separation [16,17].

Electromigration is controlled by the membrane charge density and charge polarity, which are both characterized by the zeta potential (ZP) of the membrane surface. This parameter is usually evaluated from streaming potential analyses [12,18]. The solution pH has a significant effect on ZP, because it dictates the charge on the functional groups of the membrane material and of the molecules in solution [12,16]. Moreover the pH of the system may affect the “openness”, *i.e.*, pore size, of the membrane [16], thus impacting on the size exclusion rejection mechanism.

The solution pH at which the net membrane charge is zero is the iso-electric point (IEP). The membrane surface is negatively charged, *i.e.*, negative ZP, when the solution pH is higher than the IEP and positively charged otherwise. Previous work has been carried out to determine the ZP and IEPs across a range of commercially available NF membranes. These studies were conducted for a range of single and binary salt solutions and pH values, and the IEPs of some commercially available NF membranes are summarized in Table 1. Artug and Hapke [19] determined the IEPs of three NF membranes (NF PES 10, NF 2, NF 270) as being less than three, and indicated that the distribution of dissociable acidic groups on the membrane surface, such as carboxylic and amine containing groups, determined the zeta potential of the membrane itself. Carvalho *et al.* [12] conducted tangential streaming potential (TSP) analyses on four commercially available NF membranes. They found the IEPs at pH values between five and six by using 0.1 mM KCl solutions. Their results differ from experiments conducted by other authors who tested the same NF membranes, but with different solution chemistries (10 mM NaCl). This discrepancy demonstrates that the ZP and IEP vary with solution chemistry. Childress and Elimelech [20] conducted a series of streaming potential analyses to investigate the effect of solution chemistry on the surface charge and the ZP of selected RO and NF membranes. In the presence of an electrolyte solution (NaCl), the IEP of these membranes ranged from 3.0 to 5.2, and all membranes displayed a curve characteristic of amphoteric surfaces with acidic and basic functional groups. Results with salts containing divalent ions (CaCl_2_, Na_2_SO_4_ and MgSO_4_) showed that solution chemistry has a marked effect on the surface charge, with divalent cations more readily adsorbed to the membrane surface than divalent anions.

**Table 1:** Iso-electric point (IEP) of different commercial nanofiltration (NF) membranes as measured in the existing literature.

Authors	Membrane	pH range	Solution	IEP
Childress and Elimelech [20]	NF 70	2–9	0.01 M NaCl	4
			0.01 M NaCl + 0.001 M CaCl_2_	3–3.5
			0.01 M NaCl + 0.001 M Na_2_SO_4_	4
			0.01 M NaCl + 0.001 M MgSO_4_	–
	TFCS	2–9	0.01 M NaCl	3
			0.01 M NaCl + 0.001 M CaCl_2_	3.5
			0.01 M NaCl + 0.001 M Na_2_SO_4_	3
			0.01 M NaCl + 0.001 M MgSO_4_	3
Hagmeyer and Gimbel [21]	Desal 5 DK	3–11	0.002 M KCl	4
	NTR-729	3–11	0.002 M KCl	4
Childress and Elimelech [16]	NF 55	3–9	0.01 M NaCl	3.2
			0.01 M NaCl + 2 mg L^−1^ humic acids	no IEP
			0.01 M NaCl + 1 mM surfactants	no IEP
Tanninen *et al.* [22]	NF 270	–	0.001 M KCl	3.3
	Desal 5 DK	–	0.001 M KCl	4.1
	Desal KH	–	0.001 M KCl	4.9
	BTP-NF-1	–	0.001 M KCl	6
	BTP-NF-2	–	0.001 M KCl	5.4
Artug [15]	NF 270	2.5–7	0.001 M NaCl	2.8
			0.001 M CaCl_2_	3.5
	NF 90	2.5–7	0.001 M NaCl	4.3
			0.001 M CaCl_2_	4.3
	NF PES 10	2.5–7	0.001 M NaCl	3.4
			0.001 M CaCl_2_	3.5
	NF 2	2.5–7	0.001 M NaCl	3.2
			0.001 M CaCl_2_	2.9

A thorough understanding of the membrane performance (*i.e.*, water flux and solute rejection) as a function of feed pH is mandatory, because pH affects several of the system characteristics [16]. Many studies focusing on the relationship between feed pH, membrane charge and ion rejection agree on the significant effect of feed pH, with abrupt changes and minimum rejections being expected at the IEP [15,17,21]. Minimum rejections at the IEP are explained as a consequence of the fact that size exclusion is the only active separation mechanism at the IEP [23]. In the case of a NaCl solution, Childress and Elimelech [16] found that water flux was maximal and salt rejection minimal at the membrane pore IEP, primarily due to decreased electrostatic repulsion and increased pore size. Hagmeyer and Gimbel [21] used ZP measurements to predict ion rejection of two NF membranes in binary and ternary ion solutions. While for one NF membrane, minimum rejections were found at the IEP, the second NF membrane minimum of rejection was found to be at a pH value one unit higher than the IEP. Zhong *et al.* [9] also found minimum rejections at feed pH one or two pH units higher than the IEP. In their tests on NF 270 membrane, Al-Rashdi *et al.* [24] found minimum rejections at the IEP for some, but not all metals. All the reviewed papers explained the observed trends as a function of feed pH and membrane charge polarity relative to the IEP; however, no detailed explanation was given regarding the occurrence of minimum rejections, either at or above the IEP. These studies demonstrate the complexity of NF separation mechanisms and the need for further research to fully understand the performance of NF. This is particularly relevant in more complex multi-component chemical streams, such as MIW. 

Since the IEP of commercially available NF membranes ranges between pH 3 to 5 (Table 1), thus bracketing the pH range of most MIW and AMD streams, understanding the rejection behaviour for a particular membrane-mine water problem is critical for the evaluation of a NF treatment strategy. The objective of this study was to investigate the performance of two NF membranes on different MIW streams, in order to: (i) understand the relationship between solute rejection and feed pH; and (ii) determine the commercial implications associated with optimal NF membrane selection for specific mine water streams. 

## 2. Experimental Section

### 2.1. Membranes and Mine Water Samples

Two NF membranes were tested in this study. A Dow NF 270 polyamide thin film composite NF membrane was used, because of the availability of published work describing its zeta potential and IEP and, therefore, the ability to compare the current results. NF 270 is considered a “loose” NF membrane [24], with nominal MgSO_4_ rejections of about 97% and molecular weight cut-off of 270 Da. The published NF 270 IEP range is between pH 2.5 and 4 [15,22,24]. A TriSep TS 80 polyamide thin film composite NF membrane was also assessed as an example of a “tight” NF membrane. It is characterized by a nominal monovalent ion rejection of 80%–90%, a higher than 99% rejection of polyvalent ions and has a molecular weight cut-off between 100 and 200 Da. The IEP of TS 80 has been found at about pH 3 [25,26]. 

A sample of mine water was provided by a copper mine in Western Australia, and identified hereafter as MW A. The sample originated as mine runoff during periods of intense precipitation at the mine site. The composition of MW A is shown in Table 2. Two more samples, namely MW B and MW C, were recreated as based on the analytical composition of MW A. The pH of the samples (A–C) varied from 4 to 5.5 (Table 2). A fourth sample referred to as MW D was prepared by modifying the pH of sample MW C (Table 2). MW D has a very similar composition to MW C, however the pH was lowered to a value of 2.60 by titration with hydrochloric acid. 

### 2.2. Methods

Three sets of tests were conducted on four mine water samples and on two NF membranes.

#### 2.2.1. IEP Tests

A first set of tests, referred to as the IEP Tests (Table 3), were performed to empirically estimate the position of the IEP and the relative membrane charge polarity of NF 270 and TS 80 by filtration of a NaCl-Na_2_SO_4_ solution. The literature suggests that the position of the IEP and membrane charge can be estimated from the rejection minima of simple ternary ion systems [15,27]. The synthetic NaCl-Na_2_SO_4_ solution contained approximately 700 mg L^−1^ of sodium chloride and 15 g L^−1^ of sodium sulphate, within the sulphate levels of typical mine water solutions [6]. The IEP tests were carried out with feeds ranging from pH 5 to pH 2 in 0.2 pH decrements by dosing hydrochloric acid. Test details, *i.e.*, the feed flow rate, feed temperature, feed pressure and permeate flux rate, are listed in Table 3. 

**Table 2:** Composition of mine water (MW) samples. MW A: provided by a copper mine in Western Australia. MW B and MW C: samples recreated based on the analytical composition of MW A. MW D: sample prepared from MW C by titrating the pH down from 4.10 to 2.60. NM: parameter not measured.

Parameter	Unit	MW A	MW B	MW C	MW D
pH	–	4.56	5.50	4.10	2.60
Aluminium, Al^3+^	mg L^−1^	14	0.4	NM	NM
Calcium, Ca^2+^	mg L^−1^	480	260	280	270
Copper, Cu^2+^	mg L^−1^	410	270	610	590
Iron, Fe^3+^	mg L^−1^	0.14	0.02	NM	NM
Potassium, K^+^	mg L^−1^	310	340	NM	NM
Magnesium, Mg^2+^	mg L^−1^	770	870	900	900
Manganese, Mn^3+^	mg L^−1^	440	420	530	500
Sodium, Na^+^	mg L^−1^	2000	3000	3800	3600
Sulphate, SO_4_^2−^	mg L^−1^	6900	8700	10,500	10,200
Chloride, Cl^−^	mg L^−1^	2300	NM	3000	2900

**Table 3:** Details of experimental tests conducted on four mine influenced water samples and two nanofiltration membranes.

Type of Test	Feed Sample	NF Membrane	Feed Flow (L h^−1^)	Feed Temperature (°C)	Feed Pressure (bar)	Permeate Flux Rate (L m^−2^ h^−1^)
IEP Test	NaCl-Na_2_SO_4_	NF 270	200	37 ± 4.1	20 ± 0.0	130 ± 0.0
IEP Test	NaCl-Na_2_SO_4_	TS 80	225	25 ± 0.0	10 ± 0.5	33 ± 5.8
Feed pH Test	MW A	NF 270	200	25 ± 0.6	7 ± 1.2	32 ± 2.5
Feed pH Test	MW B	TS 80	225	25 ± 0.5	19 ± 2.8	35 ± 4.6
Recovery Test	MW C	TS 80	225	25 ± 1.2	23 ± 5.0	32 ± 2.0
Recovery Test	MW C	NF 270	225	25 ± 0.5	10 ± 2.7	34 ± 0.9
Recovery Test	MW D	TS 80	225	25 ± 0.5	22 ± 6.2	33 ± 1.6
Recovery Test	MW D	NF 270	225	25 ± 0.8	10 ± 1.9	34 ± 1.5

#### 2.2.2. Feed pH Tests

A second set of tests, referred to as the Feed pH Tests (Table 3), were carried out to determine the impact of feed pH and membrane charge on ion rejection when filtering mine water through two different NF membranes. MW A was filtered by using the NF 270 membrane. MW B was filtered using the TS 80 membrane. Each test started at the initial pH of the tested water (pH 4.56 and 5.50 for MW A and MW B, respectively), and the pH was decreased in 0.2 pH decrements by the addition of hydrochloric acid. Further test details, *i.e.*, the feed flow rate, feed temperature, feed pressure and permeate flux rate, are listed in Table 3. The membranes were conditioned for 30 min in contact with the feed at zero applied pressure at each pH value before applying pressure and collecting the samples.

#### 2.2.3. Recovery Tests

A third set of tests is referred to as the Recovery Tests (Table 3). The aim of these tests was to determine the impact of different feed pH on species rejection and to relate the results to discharge criteria and recovery of commodity metals, such as copper. These tests were conducted on samples MW C and MW D, identical except for their pH values: MW C had a pH equal to 4.10, while the pH of MW D was artificially altered to 2.60 using hydrochloric acid (Table 2). A maximum volumetric recovery of 70% was established for each test (the permeate volume is equal to 70% of the feed volume), and both NF 270 and TS 80 membranes were used to filter both feed solutions. The membranes were conditioned for 30 minutes before each test started. 

### 2.3. Experimental Set-Up

**Figure 1:**
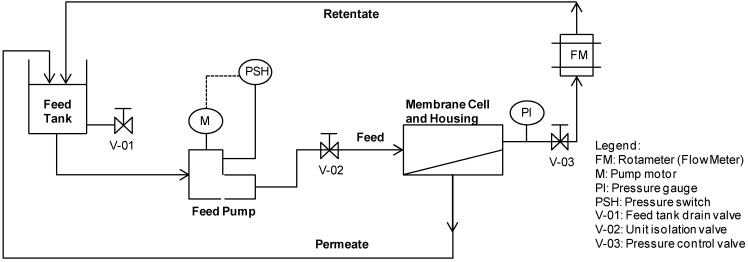
Schematic diagram of lab-scale NF unit test.

The schematic diagram of the cross-flow flat sheet membrane test unit is shown in Figure 1 (a membrane surface area of 0.0138 m^2^). Filtration experiments were carried out at operating pressures of 5 to 20 bar and a permeate flux of about 30 to 35 L m^−2^ h^−1^ (Table 3). The feed flow rate and temperature were constant at 200–225 L h^−1^ and 25 °C, respectively (Table 3). The IEP Tests and Feed pH Tests were carried out in batch re-circulation mode from a start feed volume of 2.5 L: both the permeate and retentate were re-circulated to the feed tank, except for the sample volumes of 30 mL extracted from the system at each sampling point. A composite permeate sample was collected during Recovery Tests, whilst the retentate was re-circulated to the feed tank. Feed and composite permeate samples (30 mL) were extracted at volumetric recoveries of 0%, 25%, 50%, 60% and 70%. 

All metal and sulphur analyses were conducted using inductively coupled plasma–optical emission spectroscopy (ICP-OES), while chloride analyses were conducted using an ion selective electrode. These analyses were performed by a third party commercial laboratory. A total of forty feed and permeate control samples were submitted for analysis in a number of discreet batches to determine the precision associated with the ICP-OES method. Analytical precision was calculated as the relative standard deviation of the control samples. An associated error equal to 4% was found on both feed and permeates samples. Temperature and pH were monitored during the tests using a TPS Aqua-CPA series combination pH, temperature and conductivity meter. Ion rejection was calculated for each ion as the concentration ratio between the permeate and feed sample. 

## 3. Results and Discussion

### 3.1. IEP Tests

**Figure 2:**
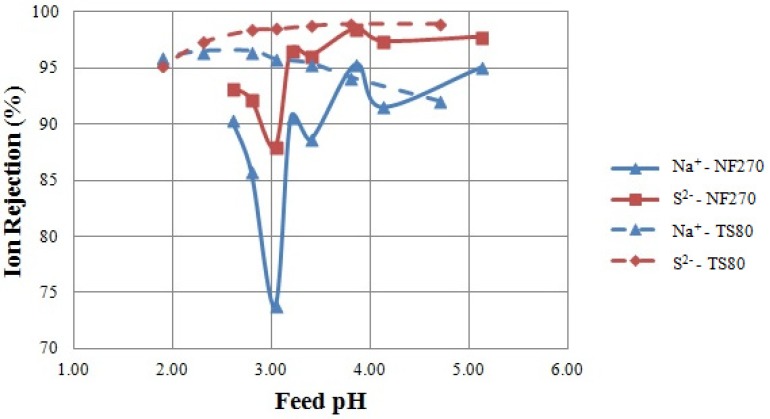
Ion rejection as a result of the IEP Tests. Tested feed solution: sodium chloride and sodium sulphate solutions. Tested membrane: NF 270 and TS 80.

The results of the IEP Tests are shown in Figure 2, where the relationship between ion rejection and feed solution pH is presented. The aim of the IEP Tests was to empirically determine the position of the IEP and the relative membrane charge polarity of NF 270 and TS 80.

Minimum rejections of sodium and sulphur were obtained at pH 3.0 when testing the NF 270 membrane (solid lines, Figure 2), suggesting that the IEP was in the vicinity of pH 3 under these conditions. This is consistent with previous studies locating the IEP of NF 270 at about pH 3 [15,22,24]. Rejection minima at the IEP were also found by Szoke *et al.* [27] and Artug [15]. It follows that in this solution, the membrane was positively charged at pH values lower than three and negatively charged at pH values higher than three. Rejections of sodium and sulphur (as sulphate) followed the same trend (solid lines, Figure 2) as expected from the maintenance of the charge balance [28]. Negative chloride rejections were observed (data not shown). This increased concentration of chloride in the permeate suggests that it passed through the membrane more easily than sulphate [22,28].

No rejection minima were found when testing the TS 80 membrane (dashed lines, Figure 2); however, a slight change in the rejection of sodium was observed around pH 3, in accordance with literature data suggesting that TS 80 has an IEP at about pH 3 [25,26]. Sodium rejections decreased at a pH higher than three (dashed lines, Figure 2), possibly explained by a negative membrane charge. In contrast, sulphate rejection increased at pH values higher than three. This trend in sulphate rejection can be explained in two ways. First, the membrane is negatively charged at a pH higher than three; thus, sulphate rejection increased in accordance with an increasingly negatively charged membrane [6,27]. Second, as the pH increases above two, sulphur is increasingly present as sulphate ion, which is highly rejected by NF membranes. As the pH is reduced, the bisulphate form predominates [22,29].

Explanations concerning the occurrence of minimum rejections at the IEP are contentious, as varying results have been observed and published in the literature. These differences are highlighted in this study, where definite rejection minima were observed for only one of two tested membranes. Similarly, Hagmeyer and Gimbel [21] and Al-Rashdi *et al.* [24] did not find results consistent with rejection minima at the IEP in their experiments. However, it was observed that either a rejection minimum or a change in the trend of ion rejection was coincident with a change in membrane charge polarity and the IEP location. In agreement with existing literature [24,26], the IEP Tests suggested that the IEP could be located at pH 3 for both membranes. 

### 3.2. Feed pH Tests

**Figure 3:**
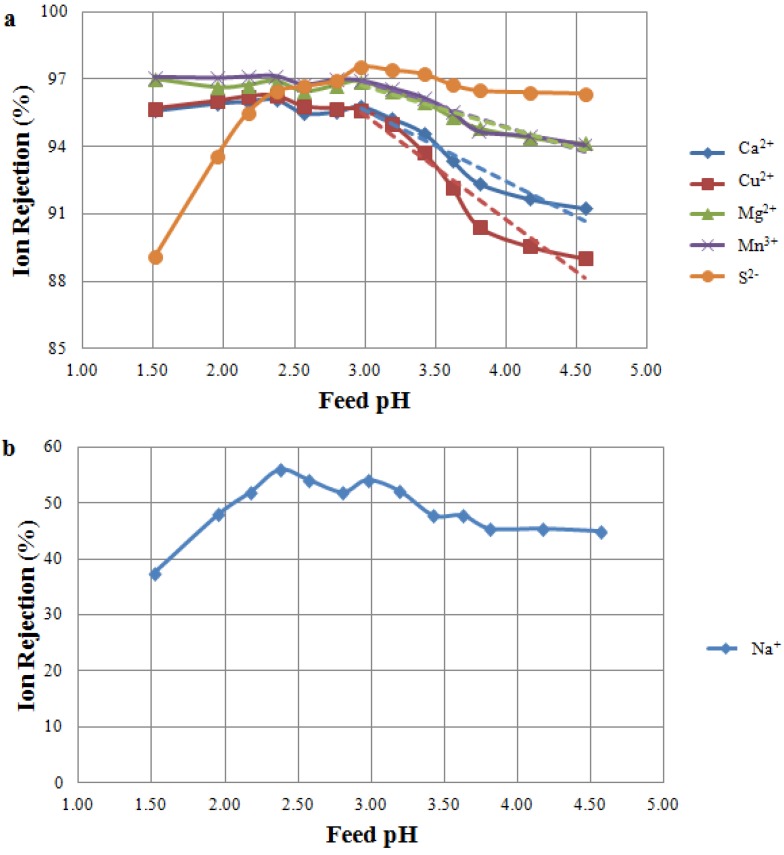
Ion rejection at varying feed pH as a result of the MW Tests. Tested feed solution: MW A. Tested membrane: NF 270 membrane. (a) Rejection of multivalent ions. Significant (p-value < 0.05) decreasing trends of cation rejections are shown for a pH higher than three; (b) Rejection of sodium ion.

**Figure 4:**
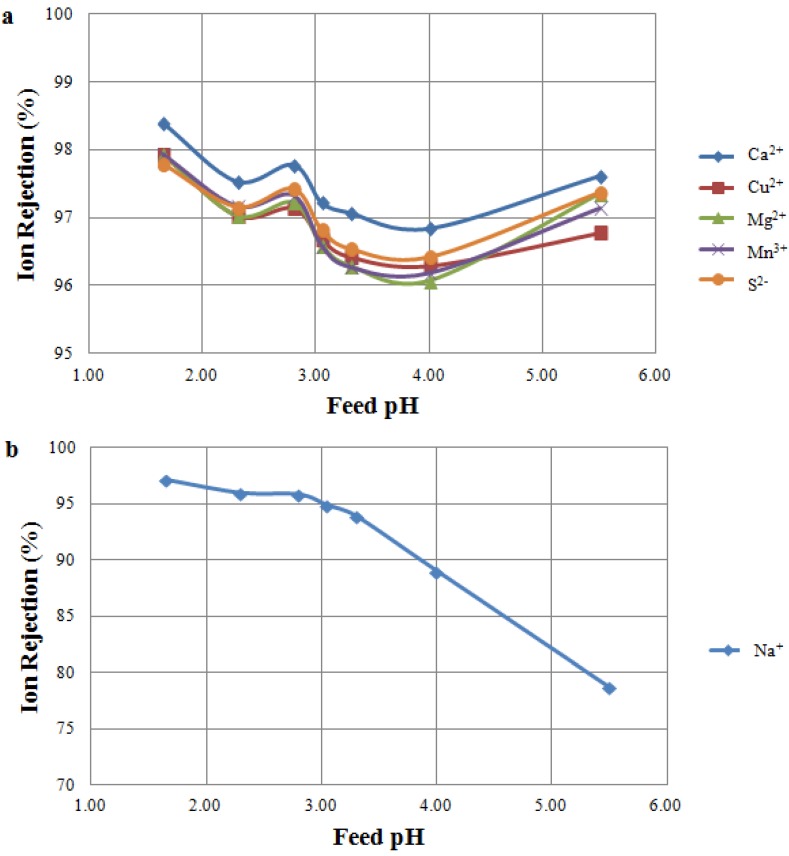
Ion rejection at varying feed pH as a result of MW Tests. Tested feed solution: MW B. Tested membrane: TS 80 membrane. (**a**) Rejection of multivalent ions; (**b**) Rejection of sodium ion.

The results of the Feed pH Tests on MW A and MW B are shown in Figure 3 and Figure 4, respectively. The objective of the Feed pH Tests was to determine the impact of feed pH on ion rejections of two different NF membranes. Note that MW A was tested on the NF 270 membrane, while MW B was tested on the TS 80 membrane. The rejections of the major cations (calcium, copper, magnesium, manganese and sodium) and sulphur (as sulphate) are shown at different feed pH values. 

#### 3.2.1. Feed pH Tests Using NF 270

Filtration of MW A through the NF 270 membrane achieved rejections above 95% for all multivalent cations at a feed pH lower than three; however, rejections decreased as the pH increased (Figure 3a). Similar trends of lower metal rejections at a high feed pH were also found by Zhong *et al.* [9] and Al-Rashdi *et al.* [24]. Cations were highly rejected when the membrane was positively charged (pH < 3), but the rejection decreased as the membrane became increasingly negative (at pH > 3). An opposite trend was observed for sulphur rejections, with higher rejection at increasing pHs (Figure 3a). This trend is explained by the joint effect of membrane charge (changing from positive to negative when passing the IEP at pH 3) and sulphate-bisulphate equilibrium. No distinct rejection minima were observed in Figure 3a for either cations or anions; however, the trends in ion rejection indicate a change in membrane function, suggesting that the IEP is in the vicinity of pH 3. As expected, sodium rejections were quite low for the NF 270 membrane, ranging between 40% and 50% (Figure 3b). 

The results obtained using the NF 270 membrane confirmed the findings of previous studies on the importance of membrane charge to determine ion rejections in NF-MIW applications [6,9,24]. NF is widely regarded as being a suitable technique to treat mine water, as it allows the concentration and recovery of valuable metals; however, the position of the membrane IEP relative to the feed pH must be carefully considered. It should be noted that NF membranes vary in terms of their rejection characteristics, and metal rejection might be further improved by deploying alternative commercially available NF membranes. 

#### 3.2.2. Feed pH Tests Using TS 80

Filtration of MW B using a TS 80 membrane showed different results when compared to the tests using NF 270 (Figure 4). Rejection of multivalent cations and sulphur was less affected by the feed pH, and rejections higher than 95% were observed across the pH range tested (Figure 4a). Moreover, as expected, higher rejections were observed for TS 80 when compared to NF 270, particularly for sulphur, copper and calcium. This is in accordance with TS 80 exhibiting a higher salt rejection than the NF 270. MacNaughton *et al.* [30] also observed very high ion rejections when comparing TS 80 with other NF membranes. Sodium rejections in the Feed pH Tests and the IEP Tests on TS 80 showed very similar results (Figure 2 and Figure 4b): in both cases, sodium rejections decreased at a pH higher than the IEP, as a consequence of the membrane charge becoming increasingly negative. 

#### 3.2.3. Comparison between NF Membranes

The results of the Feed pH Tests revealed TS 80 to be a more suitable membrane than NF 270 for treating mine water streams. Rejection performance of TS 80 was less affected by the feed pH for all multivalent cations and sulphur (Figure 3a and Figure 4a). Moreover, due to higher rejections, the use of TS 80 will maximize the concentration and recovery of commodity metals at any operating pH. It should be noted, however, that to achieve a similar flux during these tests, the TS 80 operated at almost three times the pressure of NF 270 (Table 3). This directly correlates to power consumption, giving NF 270 an advantage in terms of operating costs.

### 3.3. Recovery Tests

The results of the Recovery Tests are shown in Table 4. These tests were performed at two pH values: the recovery test on MW C was run at pH 4.10, while the recovery test on MW D was run at pH 2.60.

**Table 4:** The results of the Recovery Tests. Tested feed solution: MW D at pH 2.60 and MW C at pH 4.10. Tested membrane: NF 270 and TS 80.

Ion	Recovery Test on MW D (feed pH = 2.60; recovery = 70%)			Recovery Test on MW C (feed pH = 4.10; recovery = 70%)		Discharge Criteria (mg L^−1^)	Estimated permeate concentration second pass (mg L^−1^)	
	Rejection (%)	Permeate Concentration (mg L^−1^)		Rejection (%)	Permeate Concentration (mg L^−1^)		Feed pH = 2.60	Feed pH = 4.10
	TS 80							
Ca^2+^	98	5.7		95	13	50	0.1	0.6
Cu^2+^	97	15		94	34	1–50	0.4	1.9
Mg^2+^	97	28		94	53	50	0.9	3.1
Mn^3+^	97	13		95	29	0.005–0.5	0.3	1.6
Na^+^	94	200		87	490	–	11	63
SO_4_^2−^	98	246		95	510	250–1000	6	25
Cl^−^	84	470		78	650	–	76	141
	NF 270							
Ca^2+^	94	12		93	19	50	0.7	1.3
Cu^2+^	94	27		91	47	1–50	1.7	4.3
Mg^2+^	95	38		95	49	50	1.9	2.6
Mn^3+^	95	20		94	27	0.005–0.5	1.0	1.6
Na^+^	52	1300		50	1600	–	626	800
SO_4_^2−^	94	480		95	450	250–1000	31	22
Cl^−^	4	2200		−8	2800	–	2104	3015

#### 3.3.1. Metal Rejections at 70% Water Recovery

Ion rejections and concentrations in the composite permeates at a volumetric recovery of 70% are shown in Table 4 for the major cations (calcium, copper, magnesium, manganese and sodium) and for sulphate and chloride. Rejections were higher for all cations at a solution pH of 2.60, translating into lower ion concentrations in the composite permeate at 70% recovery (Table 4). At pH values lower than the IEP, both the NF 270 and TS 80 membranes are positively charged, and this can explain the higher rejections of cations. In order to maintain electroneutrality, the anions, sulphate and chloride, were also more rejected at pH 2.60 than at pH 4.10. These results are in accordance with the Feed pH Tests conducted with the same membranes and similar feeds. Interestingly, Na rejections are much higher for TS 80 than for NF 270, confirming TS 80’s higher published salt rejection characteristics (Table 4). The higher transmission of Na through NF 270 also explains the low and negative rejections of Cl in order to maintain electroneutrality. 

The analytical error associated with the measurement of ion concentrations in the feed and permeate samples was propagated to the calculation of ion rejection and transmission through the membranes. The precision associated with rejection data varied between 0.4% and 0.6% for all ions. The analytical error was therefore well below the difference in ion rejections at the two pH values (Table 4), confirming the difference in pH as being the main explanation of the observed changes in ion rejections. 

To the best of our knowledge, few studies have tested TS 80 on mine waters. MacNaughton *et al.* [30] tested TS 80 and other commercially available NF membranes on uranium mill effluent. The authors reported a feed pH close to neutral and stated that TS 80 was negatively charged, *i.e.*, feed pH was higher than the membrane IEP. At a volumetric recovery of 80%, they found lower rejections than in our study (rejections of Ca^2+^ ~ 85%, Mg^2+^ ~ 86%, Na^+^ ~ 25%, Mn^3+^ ~ 90%, SO_4_^2−^ ~ 85%). A comparison between the results of MacNaughton *et al.* [30] and data from the current study at feed pH 4.10 shows the importance of the feed pH and the membrane IEP in rejection performance. A general consideration for all ions in solution is that lower rejections were achieved when the feed pH was higher than the membrane IEP. 

#### 3.3.2. Nanofiltration of MIW for Environmental Discharge

Discharge criteria for mine waters are site-specific, and the industry must comply with increasingly stringent environmental targets. The application of NF as an end-of-pipe membrane treatment process to meet discharge criteria is quite well established in the literature [11,31]. General discharge criteria for water, as suggested by Rieger *et al.* [10] and shown in Table 4, were considered. 

Lower metal ion concentrations were observed in the composite permeate at pH 2.60 compared to pH 4.10 for both membranes; however, discharge limits were not met for copper or manganese in a single pass. Sulphate concentrations in the composite permeate exceeded the guideline limit at both pH values for NF 270 and at pH 4.10 for TS 80 (Table 4). In order to meet discharge criteria for all ions, a two-pass system, where the permeate from the first pass is re-filtered through a membrane, might be necessary. The concentration of ions in the permeate after a second pass was estimated assuming ion rejections remained constant for the second pass (Table 4). With a two-pass system, discharge criteria were met for sulphate at both pH values and by both membranes. However, the general discharge criteria for copper and manganese were met for TS 80 at pH 2.60 only (Table 4).

These results demonstrate that two factors need to be considered when treating mine influenced water by nanofiltration to meet discharge criteria. First, a membrane with appropriate ion rejection selectivity needs to be chosen; it was demonstrated that TS 80 offers higher rejections overall compared to NF 270 and would, therefore, be a more appropriate membrane when the ultimate treatment requirement is to meet environmental guidelines. Second, once the fit for purpose membrane is chosen, understanding the interaction between the membrane IEP and mine water pH is also important to meet discharge criteria. The guiding factor in designing a treatment for MIW is the nature of the stream to be treated, particularly the pH, the identity of the metals contained in the stream and their particular discharge criteria. 

#### 3.3.3. Nanofiltration of MIW for Metal Recovery

It has been demonstrated that NF is a viable technology in mining processes for acid and metal recovery applications [29,32,33]. NF has also been applied to the treatment of MIW [6,9,11]; however, to the best of our knowledge, few studies focused on the use of NF with the final purpose of recovering commodity metals from mine influenced water streams. 

**Figure 5:**
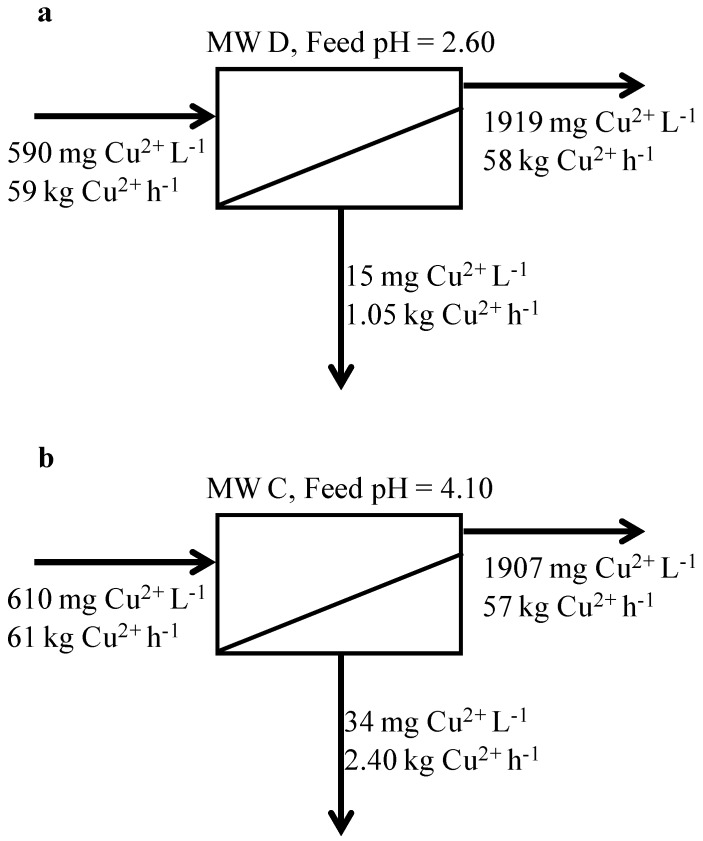
Copper mass balance calculated for (**a**) MW D at pH = 2.60 and for (**b**) MW C at pH = 4.10. The feed flow rate and volumetric recovery are fixed at 100 kL h^−1^ and 70%, respectively.

To this end, a mass balance on copper was calculated on both MW C and MW D when the TS 80 membrane was tested (Figure 5). The mass balance was based on analyses of the initial feed sample, final concentrate and composite permeate samples at 70% volumetric recovery. A feed flow rate of 100 kL h^−1^ was considered as representative of a typical mine water treatment plant. Mass balance results show that, when operating at pH higher than the IEP (pH of 4.10, Figure 5b), approximately 2.4 kg h^−1^ of copper were lost in the permeate, while only 1 kg h^−1^ was transmitted to the permeate at a pH lower than the IEP (pH of 2.60, Figure 5a). A difference of about 1.4 kg h^−1^ of copper was therefore not recovered in the concentrate when operating at a pH higher than the IEP. Given the copper price of US$5,600 per ton of CuS concentrate, this difference equates to a potential loss of Cu of about $69,000 per year. This loss could be significant in offsetting capital and operating costs and demonstrates the importance of understanding the interactions between membrane and solution chemistry.

## 4. Conclusions

The performance of two nanofiltration membranes treating mine influenced water streams was investigated in this study. Particular attention was given to the relationship between feed pH, membrane surface charge and the iso-electric point and how such a relationship impacted on ion rejections. The results were presented and discussed with the perspective of nanofiltration technology as both an end-of-pipe treatment of mine influenced water, *i.e.*, to meet environmental targets for safe discharge, and of nanofiltration technology as an in-process treatment of mine influenced water, *i.e.*, to recover valuable commodity metals, such as copper.

Ion rejection was significantly impacted by membrane charge. Metal rejection increased when the solution pH was below the membrane iso-electric point and diminished as the feed solution pH rose above the IEP, particularly when a “loose” nanofiltration membrane was used. “Tight” nanofiltration membranes with an iso-electric point higher than the feed pH can simultaneously ensure compliance with environmental guidelines and maximize copper recovery. 

Nanofiltration was shown to be successful in achieving metal recovery objectives and meeting discharge criteria; however, understanding the relationship between membrane performance and solution characteristics is essential for an optimal implementation of NF on mine influenced water.

Current research is focused on further validation of the results of this study. Additional tests are being performed with different mine water feeds and nanofiltration membranes, and a detailed cost benefit analysis is planned at the end of the test campaign, which will better quantify the lifecycle cost differences between RO and NF for a specific feed. 
